# Editorial: Lipids in immunometabolism

**DOI:** 10.3389/fimmu.2026.1812630

**Published:** 2026-02-27

**Authors:** Francesco Nicoli, Anshu Agrawal, Fabrizia Bonacina, Gareth S. D. Purvis

**Affiliations:** 1 Department of Environment and Prevention Sciences, University of Ferrara, Ferrara, Italy; 2 Division of Basic and Clinical Immunology, Department of Medicine, University of California Irvine, Irvine, CA, United States; 3 Department of Pharmacological and Biomolecular Sciences “Rodolfo Paoletti”, University of Milan, Milan, Italy; 4 Division of Cardiovascular Medicine, Radcliff Department of Medicine, University of Oxford, Oxford, United Kingdom

**Keywords:** immunometabolism, lipid metabolism, macrophage polarization, metabolic diseases, sphingolipids, T cell metabolism

A paradigm shift is underway in our understanding of lipids. Once regarded primarily as passive structural components and energy stores, lipids are now recognized as active regulators of immune cell fate and signalling. In this special Research Topic, we bring together a series of primary research articles and reviews that illuminate this rapidly evolving field and underscore the central role of lipid metabolism in immune regulation.

We begin with T cell biology, where differentiation, quiescence, activation, and polarization are tightly orchestrated by distinct bioenergetic programs. Kanno et al. provide an updated overview of these paradigms, integrating established concepts with recent multi-omics discoveries. Lipid biosynthesis offers a compelling example. Although its importance during T cell activation has long been appreciated, deeper analyses now show that these anabolic pathways are specifically directed toward the production of selected sphingolipids essential for membrane integrity and signal transduction. Transcriptomic and proteomic approaches revealed the upregulation of key biosynthetic enzymes, while lipidomic profiling identified lineage-specific enrichment of particular sphingolipid species.

Focusing on a specific T cell subset, Peesari and McAleer examine Th9 cells, producers of interleukin-9 (IL-9), a cytokine implicated in inflammatory diseases, cancer, and autoimmunity. They describe how transcription factors and lipid metabolic regulators—including mTOR, PPAR-γ, and acetyl-CoA carboxylase 1 (ACC1)—govern IL-9 production, highlighting potential therapeutic opportunities. Importantly, they emphasize the need for cell-specific targeting strategies to avoid the broad side effects that may arise from inhibiting central components of lipid metabolism. Complementing this perspective, Ertl provides a comprehensive review of T cell metabolism within the tumour microenvironment, illustrating how fluctuations in substrate availability drive metabolic adaptation not only in tumour cells but also in infiltrating T cells.

Cui et al. offers a systematic overview of lipid metabolism across immune cell types, emphasizing that metabolic programs are highly dependent on activation state and cellular identity. Each immune cell population employs distinct metabolic pathways to support its specialized functions. Drawing on examples from autoimmune diseases—including multiple sclerosis, rheumatoid arthritis, psoriasis, systemic lupus erythematosus, and inflammatory bowel disease—the review illustrates how tissue niches shape substrate availability and, consequently, immune cell function. Disease state further modifies metabolic capacity, reinforcing the dynamic interplay between lipid metabolism and immune pathology.

Turning to the innate immune system, the interplay between lipid metabolism and macrophage biology emerges as a central determinant of their cellular and systemic homeostasis. Rueda-Munguía et al. examine the multifaceted role of fatty acids in modulating macrophage phenotype and function. Fatty acids selectively engage pattern recognition receptors, triggering intracellular signalling cascades that rewire cellular energetics and shape macrophage polarization. Expanding this discussion, Rodríguez et al. consider additional lipid species, including oxylipins and phospholipids, demonstrating that lipids function not only as structural components but also as signalling mediators that influence macrophage behaviour beyond metabolic regulation alone. Together, these studies reveal a complex interdependence between macrophage polarization and lipid phenotype, underscoring the value of integrating lipidomics into multi-omic profiling approaches to uncover novel therapeutic strategies for immune-mediated and inflammation-related disorders, including inflammaging.

Collectively, the contributions in this Research Topic highlight how altered lipid metabolism and specific lipid species influence immune dysfunction and disease progression across diverse conditions, including metabolic dysfunction-associated fatty liver disease (MAFLD), obesity-related immune impairment, and chronic endometritis. In a large two-stage Chinese study, Zhao et al. showed that in MAFLD, the liver’s central role in lipid handling means that lipid accumulation provokes systemic inflammation; composite inflammatory markers linked to dysregulated lipid metabolism predict disease risk beyond traditional metabolic parameters, emphasizing that lipid-associated inflammation is integral to pathogenesis. Wilkin et al. demonstrated that in obesity, peripheral invariant NKT (iNKT) cells exhibit activation and dysfunction driven by metabolic stress, reflecting broader lipid-mediated immune perturbations characteristic of obese states. Importantly, Matsuda et al. showed that in chronic endometritis, dysregulation of SREBP1—a master regulator of lipid biosynthesis—reduces polyunsaturated fatty acids such as EPA in endometrial phospholipids, perpetuating inflammation and increasing miscarriage risk. Notably, dietary EPA supplementation restores lipid balance and mitigates inflammatory pathology, directly linking lipid composition to immune resolution and reproductive outcomes.

Taken together, the articles in this Research Topic firmly establish lipids as active regulators of immune cell fate and systemic homeostasis. While targeting anabolic and catabolic pathways offers promising therapeutic avenues, the widespread expression of metabolic enzymes across tissues demands precision-based strategies to minimize off-target effects. As the field of immunometabolism continues to mature, integrating lipid-focused approaches will be essential for translating mechanistic insight into clinical benefit.

